# Australian *Aedes aegypti* mosquitoes are susceptible to infection with a highly divergent and sylvatic strain of dengue virus type 2 but are unlikely to transmit it

**DOI:** 10.1186/s13071-020-04091-5

**Published:** 2020-05-11

**Authors:** Paul Pickering, Leon E. Hugo, Gregor J. Devine, John G. Aaskov, Wenjun Liu

**Affiliations:** 1 Australian Defence Force Malaria and Infectious Disease Institute, Brisbane, Australia; 2 Queensland Institute of Medical Research-Berghofer Medical Research Institute, Brisbane, Australia; 3 Queensland University of Technology, Brisbane, Australia

**Keywords:** *Aedes aegypti*, Dengue virus, Sylvatic transmission, Vector competence

## Abstract

**Background:**

Humans are the primary hosts of dengue viruses (DENV). However, sylvatic cycles of transmission can occur among non-human primates and human encroachment into forested regions can be a source of emergence of new strains such as the highly divergent and sylvatic strain of DENV2, QML22, recovered from a dengue fever patient returning to Australia from Borneo. The objective of the present study was to evaluate the vector competence of Australian *Aedes aegypti* mosquitoes for this virus.

**Methods:**

Four- to five-day-old mosquitoes from two strains of *Ae. aegypti* from Queensland, Australia, were fed a meal of sheep blood containing 10^8^ 50% cell culture infectious dose per ml (CCID_50_/ml) of either QML22 or an epidemic strain of DENV serotype 2 (QML16) isolated from a dengue fever patient in Australia in 2015. Mosquitoes were maintained at 28 °C, 75% relative humidity and sampled 7, 10 and 14 days post-infection (dpi). Live virions in mosquito bodies (abdomen/thorax), legs and wings and saliva expectorates from individual mosquitoes were quantified using a cell culture enzyme-linked immunosorbent assay (CCELISA) to determine infection, dissemination and transmission rates.

**Results:**

The infection and dissemination rates of the sylvatic DENV2 strain, QML22, were significantly lower than that for QML16. While the titres of virus in the bodies of mosquitoes infected with either of these viruses were similar, titres in legs and wings were significantly lower in mosquitoes infected with QML22 at most time points although they reached similar levels by 14 dpi. QML16 was detected in 16% (*n* = 25) and 28% (*n* = 25) of saliva expectorates at 10 and 14 dpi, respectively. In contrast, no virus was detected in the saliva expectorates of QML22 infected mosquitoes.

**Conclusions:**

Australia urban/peri-urban *Ae. aegypti* species are susceptible to infection by the sylvatic and highly divergent DENV 2 QML22 but replication of QML22 is attenuated relative to the contemporary strain, QML16. A salivary gland infection or escape barrier may be acting to prevent infection of saliva and would prevent onward transmission of this highly divergent virus in Australia.
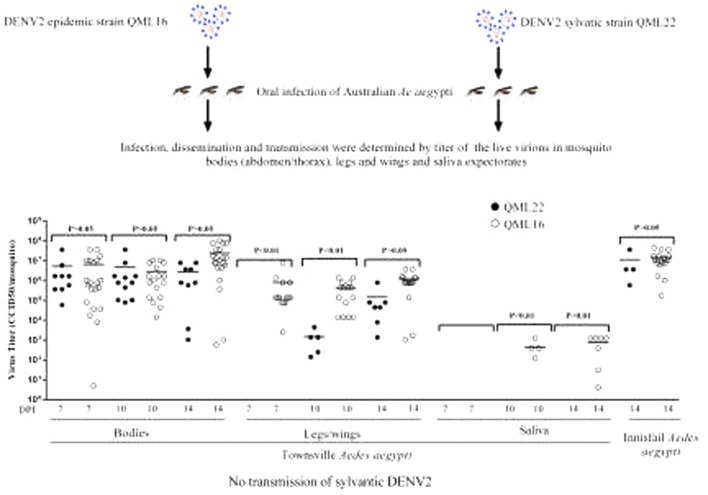

## Background

Dengue viruses (DENV) have two ecologically and evolutionarily discrete transmission cycles, sylvatic and urban endemic/epidemic [1]. The sylvatic cycle employs non-human primates as hosts and several arboreal *Aedes* mosquito species as transmission vectors [2, 3]. In contrast, the urban endemic/epidemic cycle has humans as the host and the peridomestic *Aedes aegypti* mosquito as the principal vector. All four serotypes of endemic/epidemic DENV are considered to have evolved independently from sylvatic DENV progenitors over the past 1000 years. Whether sylvatic DENV strains can overcome adaptive barriers to infect peridomestic *Ae. aegypti* mosquitoes, then enter the urban human-mosquito-human transmission cycle to cause secondary human infection (spillover epidemics), has been a source of debate for more than a decade [1, 4–6]. DENV1-4 strains from Malaysia and DENV2 strain from West Africa have been reported to spillover from sylvatic cycles to infect humans causing similar, or milder, symptoms than those caused by the classic endemic/epidemic DENV [7–11]. Previous assessments of the ability of sylvatic DENV strains to infect *Ae. aegypti* have produced a confusing picture in which the susceptibility of *Ae. aegypti* to infection with sylvatic DENV2 has ranged from refractory to almost 100% [12–15]. Significantly, none of the viruses studied were recovered from patients, instead, sylvatic viruses had been isolated from non-human primates and/or mosquitoes. In addition, these studies used virus dissemination to mosquito legs, wings and heads as a proxy for virus transmission, based on the assumption that if the virus were able to disseminate from midgut to other tissues, it would have infected the salivary glands and transmission would occur [15, 16]. The detection of infectious virus in mosquito saliva provides a more accurate proxy for transmission [17].

In 2016, a sylvatic strain of DENV2, QML22, was recovered from a patient returning to Australia from Borneo. This virus was basal to all other strains of DENV in phylogenetic trees and was divergent from Asian and West African lineages of sylvatic DENV2 [8]. Susceptibility of Australian strains of *Ae. aegypti* to infection with DENV varies with the geographical locations from which the mosquitoes are obtained [18–20]. This study determined whether colonies of *Ae. aegypti* derived from two different locations in northern Australia, where dengue outbreaks have occurred, were likely to be able to transmit this sylvatic strain of DENV if it were to be introduced.

## Methods

### Cells, viruses and mosquitoes

C6-36 (*Ae. albopictus* mosquito) cells were purchased from the American Type Culture Collection (ATCC) and cultured in 10% v/v heat-inactivated foetal calf serum (FCS, Life Technologies, Carlsbad, CA, USA)/RPMI 1640 medium (Sigma-Aldrich, St. Louis, MO, USA). The QML16 strain of DENV2 was isolated from a dengue fever patient in Australia and strain QML22 was isolated from a dengue fever patient returning to Australia from Borneo [8]. The virus strains were passaged three times in C6-36 cells and the cell culture supernatant was harvested, aliquoted and stored at – 80 °C for further use. One vial of the viral stocks was thawed to determine virus titre (CCID_50_/ml) using a cell culture enzyme-linked immunosorbent assay (CCELISA) method. As required, remaining vials were removed from the – 80 °C freezer immediately thawed, diluted and mixed with blood to prepare artificial viremic blood meals as described previously [12].

Colonies of *Ae. aegypti* were established from collections in Townsville and Innisfail in north-east Australia and maintained within the Australian Defence Force Malaria and Infectious Disease Institute and QIMR Berghofer Medical Research Institute insectaries, respectively. Both mosquito colonies were established before the release of *Wolbachia* in northern Australia. Larvae were reared at a density of 200 larvae in 3 liters of water, prepared by reverse osmosis, in plastic trays (48 × 40 × 7 cm) and fed ground TetraMin tropical fish food flakes (Tetra, Melle, Germany) at a rate of 0.25–1.00 mg/larva/day as development progressed. Pupae were transferred to cages (30 × 30 × 30 cm) for adult emergence. Adults were provided with 10% w/v sucrose solution on cotton wool pledgets which were removed 24 h prior to feeding.

### Membrane feeding

Approximately one hundred 4–5 day-old mosquitoes were placed into 750 ml containers with gauze covering the opening. Stocks of DENV2 QML16 and QML22 were thawed and immediately mixed with defibrinated sheep blood to contain 10^8^ CCID_50_/ml. The mosquitoes, in containers, were allowed to feed for 1 h on the blood/virus mixtures through bovine caecum membrane using an artificial feeding apparatus maintained at 37 °C, as previously described [21]. After feeding, mosquitoes were anaesthetized using CO_2_, placed on a Petri dish on ice and fully engorged females were separated from unfed or partially fed mosquitoes. The engorged mosquitoes were placed into the gauze covered containers, provided with cotton balls soaked with 10% sugar solution, and maintained within an environmental chamber (PHCbi, PA, USA) set at 28 °C, 75% relative humidity and 12:12 h day:night light schedule with 30 min dawn:dusk periods.

### *In vitro* transmission assays

At 7, 10 and 14 days post-infection (dpi), female mosquitoes were anesthetized using CO_2_ and placed in Petri dishes on ice. Legs and wings were removed, and their virus content used to determine the dissemination rate as described previously [22]. *In vitro* transmission assays were performed as previously described [23, 24]. For each mosquito, the proboscis was placed in a capillary tube containing 20 µl of a 1:1 solution of 50% sucrose and FCS. After 30 min, the contents were expelled into 0.25 ml MD (MD, 2% v/v FCS in RPMI 1640, 50 µg/ml penicillin/streptomycin, 50 µg/ml gentamycin, 2.5 µg/ml Amphotericin B, 10 mM HEPES) (Life Technologies). Mosquitoes were observed for abdominal contractions during the 30-min salivation period to confirm they had salivated. Those that did not appear to have salivated were discarded.

### Determination virus titre

Legs, wings and bodies from individual mosquitoes were placed into separate 2 ml screw cap vials with 1 ml MD with 4–5 zirconium silica beads. The samples were homogenized by shaking the tubes for 90 s in a chilled block using a MiniBeadbeater-96 sample homogenizer (Biospec Products, Bartlesville, OK, USA) followed by centrifugation at 17,000× *g* for 10 min at 4 °C. Supernatants were transferred to sterile tubes.

Virus stocks and virus in mosquito samples were titrated using a modification of the CCELISA procedure of Broom et al. [25]. Briefly, virus stocks and samples were 10-fold serially diluted and inoculated onto monolayers of C6/36 cells grown in RPMI 1640 supplemented with L-glutamine, 5% heat-inactivated FCS, 1% penicillin/streptomycin (Life Technologies) and maintained at 30 °C, 5% CO_2_. After 7 days of incubation, cells were fixed in acetone:methanol (1:1) for 1 h at 4 °C. Plates were air-dried and antigen was detected using a cocktail of anti-flavivirus monoclonal antibody hybridoma supernatants (4G2 [26]:6B-6C1:3H5 [27], at a ratio of 1:1:1), followed by horseradish peroxidase (HRP-) conjugated goat anti-mouse polyclonal antibody (DAKO, Carpinteria, CA, USA) (1:2000 in PBS-Tween). Antibodies bound to the cell monolayers were detected by the addition of 3,3’,5,5’-tetramethylbenzidine (TMB) Liquid Substrate System for Membranes (Sigma-Aldrich). The CCID_50_ was determined from titration endpoints as described elsewhere [28] and expressed as the C6/36 CCID_50_/ml.

The infection rate was defined as the proportion of mosquitoes with bodies containing DENV divided by the total number of engorged mosquitoes. Dissemination and transmission rates were defined as the proportions of infected mosquitoes with legs/wings containing DENV and salivary secretions containing DENV divided by the total number of engorged mosquitoes. The Mann-Whitney *U*-test, *t*-test and Chi-square tests were employed to compare virus titres in tissues and proportions of infected tissues.

### Mosquito immunohistochemistry

Histological analysis of DENV infection within mosquitoes employed indirect immunofluorescence assays (IFA) as described previously [23]. Briefly, mosquitoes with legs and wings removed were fixed in 4% v/v paraformaldehyde/0.5% v/v Triton X-100 for 12 h, dehydrated in xylol followed by a graded ethanol series, embedded in paraffin and 3–4 µM sections fixed to slides. Sections were incubated in Diva antibody retrieval solution (Biocare Medical, Concord, CA, USA) at 125 °C for 5 min in a Biocare Medical Decloaking Chamber. Sections were cooled for 20 min and washed twice in 0.025% v/v Tween 20/PBS pH 7.2 for one minute each wash. Non-specific antibody binding was inhibited by incubating the sections in 2% w/v bovine serum albumin (Sigma-Aldrich)/Biocare Medical Background Sniper for 30 min. Excess Sniper/BSA was removed from the sections before they were incubated with anti-flavivirus monoclonal antibody, 4G2, for 2 h at room temperature. Sections were washed three times with PBST and Alexa Fluor 488 donkey anti-mouse antibody diluted 1:300 in PBST applied for 30 min. Sections were washed three times with PBST before being counterstained with DAPI for 10 min, and washed 4 times with PBST before being mounted.

## Results

**Fig. 1:**
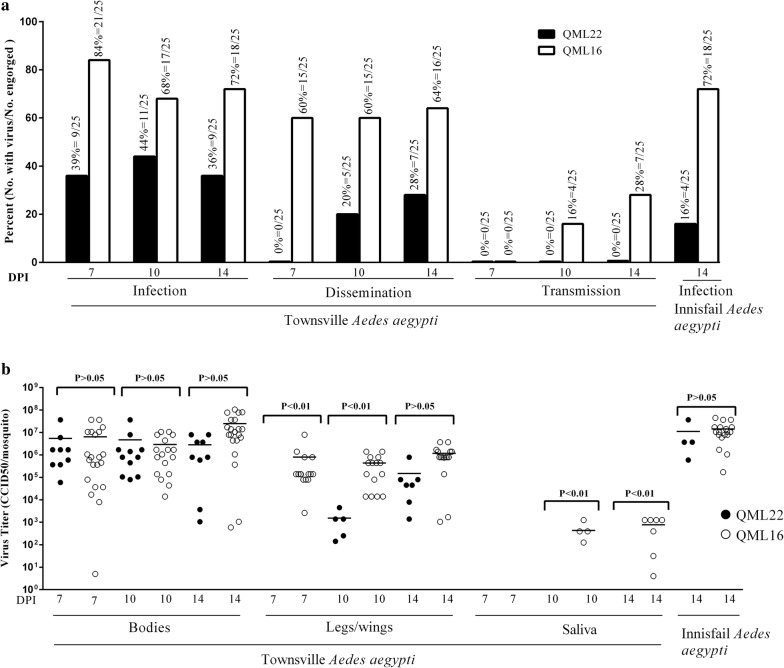
Infection rate, dissemination and transmission potential of DENV2 QML16 (open symbols) and DENV2 QML22 (closed symbols) in *Ae. aegypti* mosquitoes. **a** Infection, dissemination and transmission rates were calculated from the proportion of blood-fed mosquitoes that developed a detectable DENV infection in the bodies, legs/wings and saliva, respectively. **b** Titres of virus in tissues from infected insects quantified by CCELISA in C6/36 mosquito cells

A smaller proportion of mosquitoes from the *Ae. aegypti* colony from Townsville became infected when fed DENV2 QML22 (38.7%, *n* = 75) than QML16 (75%, *n* = 75) (Fig. 1a, b; *χ*^2^ = 24.74, *df* = 1, *P* < 0.0001) and the proportion of bodies infected with each strain remained stable between 7 and 14 dpi. Although the proportion of mosquitoes infected with QML22 was lower than that for QML16, the titres of each virus in infected mosquitoes were not significantly different (Fig. 1b) (Mann Whitney test, *U* = 695.5, *P* = 0.1562).

DENV2 QML16 was detected in legs and wings as early as 7 dpi while QML22 was not detected in these tissues until 10 dpi. Furthermore, QML22 disseminated to legs and wings in fewer mosquitoes that QML16 and virus grew to lower titres than QML16 at 7 and 10 dpi (Fig. 1a, b; *χ*^2^ = 27.47, *df* = 1, *P* < 0.001). However, QML22 reached similar titers to QML16 in legs and wings by 14 dpi (Unpaired t-test, *P* = 0.0988, Fig. 1b).

No live virus was detected in saliva expectorates of mosquitoes fed on blood meals containing QML22 7, 10 or 14 days post-feeding. This was in contrast to mosquitoes fed on blood meals containing QML16, which resulted in 16% (4/25) and 28% (7/25) of saliva samples containing virus at day 10 and 14 dpi, respectively. The titres of DENV in these samples reached a maximum of 1250 CCID_50_/mosquito.

These investigations were repeated using a colony of *Ae. aegypti* established from mosquitoes collected at Innisfail, 250 km from Townsville several years after the Townsville colony was established. The infection rate of QML22 in *Ae. aegypti* from Innisfail fed on the same concentrations as above was lower (16%, *n* = 25 at 14 dpi) than that observed with the Townsville colony (above), while the rates of infection of mosquitoes from both *Ae. aegypti* colonies with QML16 were similar (72%, *n* = 25 for Innisfail; and 79%, *n* = 75 for Townsville; *χ*^2^ = 0.38, *P* = 0.54, Fig. 1a, b). In keeping with the results of the first experiment, the titres of virus in the infected bodies were similar in mosquitoes fed QML16 and QML22 (~ 10^7^ CCID_50_/mosquito, Fig. 1b). This suggested that the *Ae. aegypti* mosquitoes from Innisfail were less susceptible to infection with QML22 than those from Townsville. Low numbers of infected mosquitoes from Innisfail prevented statistical analyses.

**Fig. 2:**
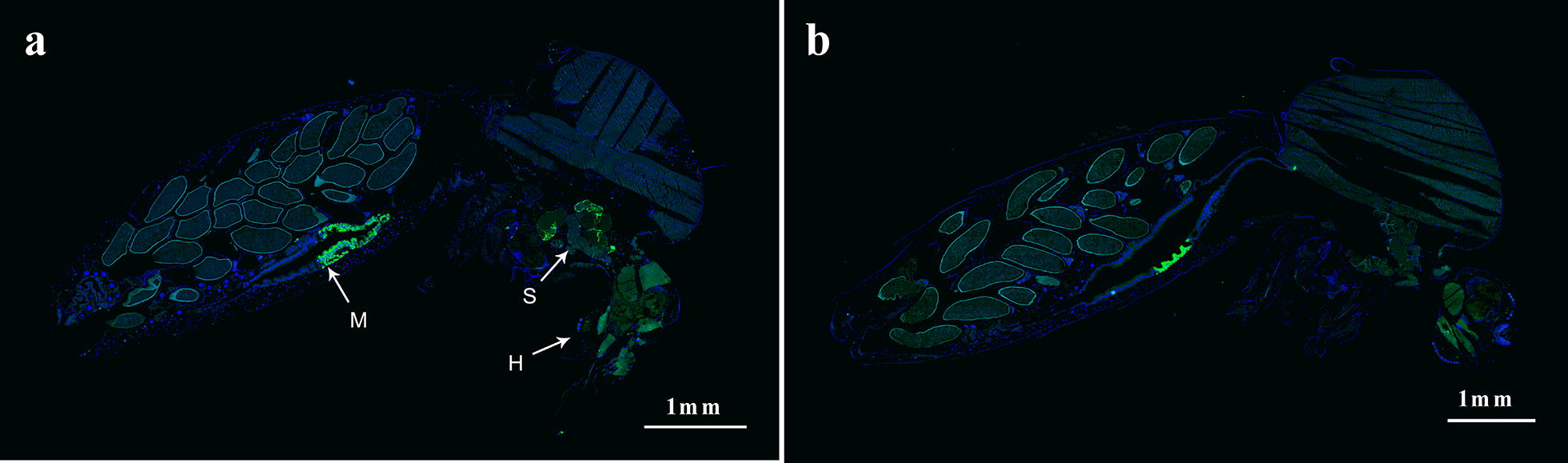
Midsagittal sections showing tissue distribution of DENV QML16 and QML22 strain infection within orally inoculated *Ae. aegypti.* Mosquitoes were examined for the distribution of DENV infection by IFA analysis of paraffin sections using an anti-flavivirus Envelope protein monoclonal antibody and Alexa-fluor 488 conjugated secondary antibody for DENV (green) and DAPI staining for DNA (blue). **a** Example of whole-body staining of mosquitoes infected with DENV QML16 at 14 dpi showing staining in head (H), midgut (M); and salivary glands (S). **b** Example of whole-body staining of mosquitoes infected with DENV QML22 at 14 dpi showing infection limited to the midgut. No staining was observed beyond midguts for mosquitoes inoculated with QML22. *Scale-bars*: 1 mm

Histological examination of limited numbers of mosquitoes infected with QML16 and QML22 supported the above results. (Fig. 2). Disseminated virus infection was observed in 88% (*n* = 25) mosquitoes ≥ 10 days after feeding on QML16 and infection was observed in 50% (*n* = 6) of the salivary glands of these insects. In contrast, no dissemination of virus could be detected beyond the midgut in any mosquitoes ≥ 10 days after feeding on QML22 (*n* = 13).

## Discussion

The introduction of a pathogenic, transmissible and highly divergent strain of DENV2 into areas of Australia with a human population largely susceptible to DENV infection as well as the existence of the principal urban vector of DENV, *Ae. aegypti* [29], could have significant public health implications. However, while colonies of *Ae. aegypti* established from two population centres in northern Australia were found to be susceptible to infection with, and able to transmit, a contemporary epidemic strain of DENV2, they were much less susceptible to infection with the highly divergent and sylvatic strain, of DENV2 QML22, and thus appear unlikely to be able to transmit it.

Variable competencies of *Ae. aegypti* populations from around the world to act as vectors for sylvatic DENV have been reported. A sylvatic strain of DENV2 isolated from a mosquito in Senegal in 1965 was found to infect 50–91% of eight different Senegalese *Ae. aegypti* populations. Moreover, these results were achieved from blood meals containing substantially less virus than used in this study (approximately 10^6–7^ PFU/ml virus) [14]. In contrast, infection rates of only 0–27% were observed in another study in which six Senegalese *Ae. aegypti* populations were fed 10^6–7^ PFU/ml of a sylvatic strain DENV2 isolated from Senegal in 1999 [12]. Our infection rates more closely resemble those achieved when *Ae. aegypti* collected from Galveston, USA, and from Bolivia were fed on blood meals containing 10^8^ to 10^9.5^ TCID_50_/ml of strains of sylvatic DENV2 isolated from a mosquito in Burkina Faso, West Africa, and from a sentinel monkey in Malaysia [30]. All these studies used dissemination to distal body tissues as a measure of the potential to transmit these viruses based on an assumption that mosquitos were capable of transmitting DENV if the virus had disseminated from the midgut into the hemocoel [15, 16]. In our experiments with QML22, the virus disseminated into legs and wings but could not be detected in saliva at any time point.

Our data reinforce the extensive DENV vector competency literature that demonstrates that no two strains of DENV can be assumed to behave in exactly the same way in *Ae. aegypti* from different localities. Investigations to determine the mechanisms underpinning the resistance of *Ae. aegypti* to infection with this sylvatic strain of DENV2 are likely to be complex given the enormous differences between the nucleotide and amino acid sequences of it and other strains of DENV2 for which *Ae. aegypti* is known to be able to be a competent vector [5]. Added to this is the additional complexity of host factors that this study observed in two colonies of mosquitoes derived from areas only 250 km apart.

In order to transmit the virus to an uninfected human, DENV must escape the mosquito innate immune system to replicate and disseminate through the mosquito before entering saliva [31]. Several physiological ‘barriers’ to this dissemination have been hypothesised, including midgut infection and escape barriers (MIB and MEB) and salivary gland infection and escape barriers (SGIB and SGEB) and earlier studies have indicated that the MIB is a major determinant of vector competence for DENV [32, 33]. The lower body infection rate of QML22 (Fig. 1a) suggested a MIB might be the first obstacle for the highly divergent QML22 where the virus/cell-receptor interaction and internalization into the midgut epithelial cells is occurring. When the MIB was overcome, QML22 replicated to titres similar to those for QML16 in body tissues (Fig. 1b). Lower dissemination rates and slower replication rates for QML22 than QML16 in legs and wings would have a significant effect on transmission given the relatively short half-life of *Ae. aegypti* in nature. Failure to detect infectious QML22 in mosquito saliva by CCELISA was not surprising given the inability to detect DENV in salivary glands from a small number of mosquitoes infected with QML22 (Fig. 2). However, the difference between the proportion of QML16 infected mosquitoes with infected salivary glands and the proportion with virus in saliva (Figs. 1 and 2), suggested the SGEB may play a role in determining the competency of Australian *Ae. aegypti* mosquitoes to transmit DENV. The relative importance of physiological infection barriers remains to be further determined.

The marked differences between the ability of colonies of *Ae. aegypti* to become infected with and to transmit this highly divergent/primitive strain of DENV 2 (QML22) and a conventional strain (QML16) suggest further studies with *An. albopictus* and arboreal strains of *Aedes* are warranted, if such colonies can be established, to determine whether other strains of *Ae. aegypti* also are poor vectors of QML22 or whether there is a gradient of competencies from arboreal to urban mosquitoes.

The use of frozen stocks of viruses for mosquito vector competence studies has been associated with a reduction in the infectivity of virus for mosquitoes compared to that of freshly prepared virus stocks [34]. However, the use of frozen stocks was an experimental requirement for robust comparisons of the two strains and both strains were treated in the same way [6, 13, 24, 35].

## Conclusions

*Aedes aegypti* mosquitoes from Townsville and Innisfail in northern Australia are highly susceptible to infection with and able to transmit a contemporary epidemic strain of DENV2 but are much less susceptible to infection with a highly divergent and sylvatic DENV2, QML22, and, potentially, are unable to transmit it. Our findings support a conclusion that sylvatic DENV is unlikely to enter urban human-mosquito-human transmission cycles in Australia [36].

## Data Availability

The data supporting the conclusions of this article are included within the article. Raw data and materials are available from the corresponding author upon request.

## Abbreviations

ATCC: American Type Culture Collection
CCELISA: cell culture enzyme-linked immunosorbent assay
CCID_50_: 50% cell culture infectious dose
DENV: dengue virus
dpi: days post-infection
FCS: foetal calf serum
HRP: horseradish peroxidase
IFA: indirect immunofluorescence assay
PBST: PBS+0.025% Tween 20
QLD: Queensland
VC: vector competence (VC)
TMB: tetramethylbenzidine

## References

[1] Vasilakis N, Cardosa J, Hanley KA, Holmes EC, Weaver SC (2011). Fever from the forest: prospects for the continued emergence of sylvatic dengue virus and its impact on public health. Nat Rev Microbiol.

[2] Vasilakis N, Holmes EC, Fokam EB, Faye O, Diallo M, Sall AA (2007). Evolutionary processes among sylvatic dengue type 2 viruses. J Virol.

[3] Vasilakis N, Fokam EB, Hanson CT, Weinberg E, Sall AA, Whitehead SS (2008). Genetic and phenotypic characterization of sylvatic dengue virus type 2 strains. Virology.

[4] Mota J, Rico-Hesse R (2009). Humanized mice show clinical signs of dengue fever according to infecting virus genotype. J Virol.

[5] Vasilakis N, Shell EJ, Fokam EB, Mason PW, Hanley KA, Estes DM (2007). Potential of ancestral sylvatic dengue-2 viruses to re-emerge. Virology.

[6] Gaye A, Wang E, Vasilakis N, Guzman H, Diallo D, Talla C (2019). Potential for sylvatic and urban *Aedes* mosquitoes from Senegal to transmit the new emerging dengue serotypes 1, 3 and 4 in West Africa. PLoS Negl Trop Dis.

[7] Carey DE, Causey OR, Reddy S, Cooke AR (1971). Dengue viruses from febrile patients in Nigeria, 1964–68. Lancet.

[8] Liu W, Pickering P, Duchene S, Holmes EC, Aaskov JG (2016). Highly divergent dengue virus Type 2 in traveler returning from Borneo to Australia. Emerg Infect Dis.

[9] Pyke AT, Moore PR, Taylor CT, Hall-Mendelin S, Cameron JN, Hewitson GR (2016). Highly divergent dengue virus type 1 genotype sets a new distance record. Sci Rep.

[10] Zeller HG, Traore-Lamizana M, Monlun E, Hervy JP, Mondo M, Digoutte JP (1992). Dengue-2 virus isolation from humans during an epizootic in southeastern Senegal in November, 1990. Res Virol.

[11] Saluzzo JF, Cornet M, Castagnet P, Rey C, Digoutte JP (1986). Isolation of dengue 2 and dengue 4 viruses from patients in Senegal. Trans R Soc Trop Med Hyg.

[12] Diallo M, Ba Y, Faye O, Soumare ML, Dia I, Sall AA (2008). Vector competence of *Aedes aegypti* populations from Senegal for sylvatic and epidemic dengue 2 virus isolated in West Africa. Trans R Soc Trop Med Hyg.

[13] Diallo M, Sall AA, Moncayo AC, Ba Y, Fernandez Z, Ortiz D (2005). Potential role of sylvatic and domestic African mosquito species in dengue emergence. Am J Trop Med Hyg.

[14] Dickson LB, Sanchez-Vargas I, Sylla M, Fleming K, Black WCt (2014). Vector competence in West African *Aedes aegypti* is *Flavivirus* species and genotype dependent. PLoS Negl Trop Dis.

[15] Moncayo AC, Fernandez Z, Ortiz D, Diallo M, Sall A, Hartman S (2004). Dengue emergence and adaptation to peridomestic mosquitoes. Emerg Infect Dis.

[16] Gubler DJ, Nalim S, Tan R, Saipan H, Sulianti Saroso J (1979). Variation in susceptibility to oral infection with dengue viruses among geographic strains of *Aedes aegypti*. Am J Trop Med Hyg.

[17] Anderson SL, Richards SL, Smartt CT (2010). A simple method for determining arbovirus transmission in mosquitoes. J Am Mosq Control Assoc.

[18] Watson TM, Kay BH (1999). Vector competence of *Aedes notoscriptus* (Diptera: Culicidae) for Barmah Forest virus and of this species and *Aedes aegypti* (Diptera: Culicidae) for dengue 1–4 viruses in Queensland, Australia. J Med Entomol.

[19] Knox TB, Kay BH, Hall RA, Ryan PA (2003). Enhanced vector competence of *Aedes aegypti* (Diptera: Culicidae) from the Torres Strait compared with mainland Australia for dengue 2 and 4 viruses. J Med Entomol.

[20] Ye YH, Ng TS, Frentiu FD, Walker T, van den Hurk AF, OʼNeill SL (2014). Comparative susceptibility of mosquito populations in North Queensland, Australia to oral infection with dengue virus. Am J Trop Med Hyg.

[21] Rutledge LC, Ward RA, Gould DJ (1964). Studies on the feeding response of mosquitoes to nutritive solutions in a new membrane feeder. Mosq News.

[22] Veronesi E, Paslaru A, Silaghi C, Tobler K, Glavinic U, Torgerson P (2018). Experimental evaluation of infection, dissemination, and transmission rates for two West Nile virus strains in European *Aedes japonicus* under a fluctuating temperature regime. Parasitol Res.

[23] Hugo LE, Prow NA, Tang B, Devine G, Suhrbier A (2016). Chikungunya virus transmission between *Aedes albopictus* and laboratory mice. Parasites Vectors.

[24] Hugo LE, Stassen L, La J, Gosden E, Ekwudu O, Winterford C (2019). Vector competence of Australian *Aedes aegypti* and *Aedes albopictus* for an epidemic strain of Zika virus. PLoS Negl Trop Dis.

[25] Broom AK, Hall RA, Johansen CA, Oliveira N, Howard MA, Lindsay MD (1998). Identification of Australian arboviruses in inoculated cell cultures using monoclonal antibodies in ELISA. Pathology.

[26] Roehrig JT, Mathews JH, Trent DW (1983). Identification of epitopes on the E glycoprotein of Saint Louis encephalitis virus using monoclonal antibodies. Virology.

[27] Henchal EA, Gentry MK, McCown JM, Brandt WE (1982). Dengue virus-specific and flavivirus group determinants identified with monoclonal antibodies by indirect immunofluorescence. Am J Trop Med Hyg.

[28] Charretier C, Saulnier A, Benair L, Armanet C, Bassard I, Daulon S (2018). Robust real-time cell analysis method for determining viral infectious titers during development of a viral vaccine production process. J Virol Methods.

[29] Naish S, Dale P, Mackenzie JS, McBride J, Mengersen K, Tong S (2014). Spatial and temporal patterns of locally-acquired dengue transmission in northern Queensland, Australia, 1993–2012. PLoS ONE.

[30] Young KI, Mundis S, Widen SG, Wood TG, Tesh RB, Cardosa J (2017). Abundance and distribution of sylvatic dengue virus vectors in three different land cover types in Sarawak, Malaysian Borneo. Parasites Vectors.

[31] Franz AW, Kantor AM, Passarelli AL, Clem RJ (2015). Tissue barriers to arbovirus infection in mosquitoes. Viruses.

[32] Mercado-Curiel RF, Black WCt, Munoz MdL (2008). A dengue receptor as possible genetic marker of vector competence in *Aedes aegypti*. BMC Microbiol.

[33] Salas-Benito J, Reyes-Del Valle J, Salas-Benito M, Ceballos-Olvera I, Mosso C, del Angel RM (2007). Evidence that the 45-kD glycoprotein, part of a putative dengue virus receptor complex in the mosquito cell line C6/36, is a heat-shock related protein. Am J Trop Med Hyg.

[34] Richards SL, Pesko K, Alto BW, Mores CN (2007). Reduced infection in mosquitoes exposed to blood meals containing previously frozen flaviviruses. Virus Res.

[35] Bennett KE, Olson KE, Munoz MdL, Fernandez-Salas I, Farfan-Ale JA, Higgs S (2002). Variation in vector competence for dengue 2 virus among 24 collections of *Aedes aegypti* from Mexico and the United States. Am J Trop Med Hyg.

[36] Vasilakis N, Weaver SC (2008). The history and evolution of human dengue emergence. Adv Virus Res.

